# Basal core promoter and precore mutations among hepatitis B virus circulating in Brazil and its association with severe forms of hepatic diseases

**DOI:** 10.1590/0074-02760160540

**Published:** 2017-09

**Authors:** Silvana Gama Florencio Chachá, Michele Soares Gomes-Gouvêa, Fernanda de Mello Malta, Sandro da Costa Ferreira, Márcia Guimarães Villanova, Fernanda Fernandes Souza, Andreza Correa Teixeira, Afonso Dinis da Costa Passos, João Renato Rebello Pinho, Ana de Lourdes Candolo Martinelli

**Affiliations:** 1 Universidade Federal de São Carlos, Departamento de Medicina, São Carlos, SP, Brasil; 2 Universidade de São Paulo, Faculdade de Medicina de Ribeirão Preto, Departamento de Clínica Médica, Divisão de Gastroenterologia, Ribeirão Preto, SP, Brasil; 3 Faculdade de Medicina da Universidade de São Paulo, Instituto de Medicina Tropical, Departamento de Gastroenterologia, Laboratório de Gastroenterologia e Hepatologia Tropical, São Paulo, SP, Brasil; 4 Universidade de São Paulo, Faculdade de Medicina de Ribeirão Preto, Departamento de Medicina Social, Ribeirão Preto, SP, Brasil; 5 Hospital Israelita Albert Einstein, São Paulo, SP, Brasil

**Keywords:** hepatitis B virus, basal core promoter mutant, precore mutant, Brazil

## Abstract

**BACKGROUND:**

In Brazil, few studies have investigated the prevalence of infection with the precore (PC) and basal core promoter (BCP) mutants of the hepatitis B virus (HBV).

**OBJECTIVES:**

This study aimed to analyse the frequency of PC and BCP mutations among patients infected with HBV and to evaluate the association between the variants and advanced hepatic disease.

**METHODS:**

A total of 161 patients infected with HBV were studied. To identify PC and BCP mutations, a 501-bp fragment of HBV DNA was amplified and sequenced.

**FINDINGS:**

PC and BCP regions from HBV strains were successfully amplified and sequenced in 129 and 118 cases, respectively. PC and BCP mutations were detected in 61.0% and 80.6% of the cases, respectively. The A1762T/G1764A variant was identified in 36.7% of the patients with grade 1 and 2 liver fibrosis (29/79) and in 81.8% of the patients with grade 3 and 4 liver fibrosis (9/11) (p < 0.01); in 76.9% of the patients with cirrhosis (10/13) and in 38.1% of the patients without cirrhosis (40/105) (p = 0.01); and in 77.8% of the patients with hepatocellular carcinoma (HCC) (7/9) and in 39.4% of the patients without HCC (43/109) (p = 0.03).

**MAIN CONCLUSIONS:**

A high prevalence of HBV PC and BCP mutants was found. The A1762T/G1764A variant was independently associated with advanced forms of liver fibrosis, hepatic cirrhosis, and HCC.

The natural course of chronic hepatitis B virus (HBV) infection involves varied forms ranging from inactive carriers to patients with cirrhosis, hepatocellular carcinoma (HCC), or liver failure (Chang 2010, McMahon 2010, Dandri & Locarnini 2012). Due to such a broad spectrum of clinical presentations, the search for factors predictive of the disease evolution has proved to be highly valuable for deciding the strategy to monitor and treat the infected patients (McMahon 2010). The study of the natural history of HBV infection has been fundamental for understanding disease evolution and for guiding the clinical management of infected patients.

Evidence has demonstrated that genetic variations in HBV can influence the pathogenesis of the disease (Kao et al. 2003, McMahon 2010). Some mutations in the basal core promoter (BCP) and precore (PC) genomic regions were found to be related to changes in viral kinetics - a reduced synthesis of the *e* antigen (HBeAg) and differences in necroinflammatory activity in the liver (Hadziyannis & Vassilopoulus 2001, Kao et al. 2003). Studies from Asian countries have demonstrated that BCP variants may be implicated in the progression of liver injury and its early progression to HCC (Yang et al. 2008, Zheng et al. 2011). However, few studies have determined the behaviour of the HBV genetic variants circulating in Brazil, particularly in the most prevalent genotypes, A, D and F, found in this geographical region, and the influence of the HBV PC and BCP variants on liver disease in the Brazilian population infected with HBV. The present study aimed to analyse the frequency of BCP and PC mutations in the HBV genome isolated from Brazilian patients, and to evaluate the association between the HBV PC and BCP mutants and the phase of HBV chronic infection, stage of liver fibrosis, and presence of cirrhosis or HCC.

## MATERIALS AND METHODS

A total of 161 patients infected with chronic HBV visiting the Hepatitis B Outpatient Clinic of the Ribeirão Preto Medical School, University of São Paulo, were recruited between July 2007 and July 2009.

The inclusion criterion was the presence of the HBsAg in the serum for more than six months, as well as agreeing to participate in the study. Exclusion criteria were the presence of the hepatitis C virus (HCV) and/or human immunodeficiency virus co-infection and metabolic or autoimmune chronic liver disease.

The study model was cross-sectional. On the day of inclusion, the patients were interviewed. A review of the medical records, laboratory tests, and imaging and endoscopic examinations from the previous year was performed. When available, liver biopsies were used for evaluation of fibrosis stages and histopathological activity index according to the classification proposed by Knodell et al. (1981) and modified by Desmet et al. (1994). Peripheral venous blood samples were collected and analysed for the detection of viral antigens (HBsAg, HBeAg) - anti-HBeAg (AxSYM, ABBOTT) and anti-HDV (ETI-AB-DELTAK-2, anti-HD, Diasorin) - by ELISA. Blood samples were collected and used for the determination of hepatitis B viral load. The viral load was used to aid in the distribution of patients into groups according to the evolutionary stage of chronic HBV infection, and was determined through absolute quantitation of HBV DNA by real-time polymerase chain reaction (PCR) using TaqMan® (Applied Biosystems), with primers and probes annealing to the regions of the S gene conserved in all HBV genotypes (Sitnik et al. 2010). No information regarding previous antiviral treatment was available.

Patients were first classified into three groups according to the phase of chronic infection - HBeAg-positive chronic hepatitis, HBeAg-negative chronic hepatitis, or inactive carrier (Lok & McMahon 2009). For further analysis, the research subjects were classified in a different way, according to the grade of liver fibrosis and according to the presence or absence of cirrhosis and/or HCC (Bruix & Sherman 2005, Bosch et al. 2008).

The diagnosis of cirrhosis was based on clinical features (ascites, spiders, encephalopathy, etc.), biochemical and coagulation tests, liver and portal system Doppler ultrasonography, and upper gastrointestinal endoscopic parameters, or the presence of characteristic histopathological features observed in a liver biopsy fragment (Desmet et al. 1994, Bosch et al. 2008). The diagnosis of HCC was defined according to the guidelines of the Barcelona-Clinic Liver Cancer Group (Llovet et al. 2004).

Collected blood samples were also used for analysis of the HBV genotypes, and PC and BCP mutations. Genotype analysis was performed by amplification of a 1306-bp fragment in the S and polymerase regions of the HBV genome (S/POL), using primers that amplify the entire reverse transcriptase region of the viral polymerase DNA gene, as previously described, and phylogenetic analysis of the sequences were performed (Gomes-Gouvêa et al. 2015).

To identify PC and BCP mutations, the HBV DNA genome region was amplified using nested-PCR. First, 2032R and EP 1.1 primers were used, generating an amplicon of 554 bp. Next, 2017R and EP 2.1 were used, generating an amplicon of 501 bp. After quantification and purification, the amplified DNA was sequenced using the primers similar to those used in the second amplification reaction, on ABI PRISM 3100. Mutations in the BCP and PC regions were identified by visual analysis of the nucleotide sequence alignment. The following positions were analysed: 1753, 1762, 1764, 1766, and 1768 in the BCP region; and 1814, 1815, 1816, 1862, 1896 and 1899 in the PC region. All nucleotides differing from thymine (T)1753, cytosine (C)1766, T1768, adenine (A)1762, guanine (G) 1764, A1814, T1815, G1816, G1862, G1869, and G1899 were considered in our analysis.

Data were analysed statistically using SPSS (IBM SPSS ® Statistics, version 17.0) software. The mean, median, and standard deviation were calculated and the Fischer Exact Test, Chi-Square Test, and non-parametric Mann-Whitney and Kruskal-Wallis tests were applied when indicated, with the level of significance set at < 0.05 in all analyses.

All the procedures followed in this study were in accordance with the Helsinki Declaration of 1975, as revised in 1983. The study was approved by the Research Ethics Committee of the Hospital (authorisation No. 1809/2007) and all the patients gave written informed consent to participate.

## RESULTS

*Study population* - The main demographic, clinical, and histological characteristics of the included patients and the evolutionary HBV disease form are shown in Table I. All included patients were anti-HDV negative.

**TABLE I:** Demographic, clinical and histological characteristics of the 161 chronic hepatitis B virus (HBV) patients included in the study according to the different evolutionary stages of chronic HBV infection: number of patients

	HBeAg positive n = 26	HBeAg negative chronic hepatitis n = 108	Inactive carrier n = 27	p
Age (years)				
mean	39.9	45.0	44.4	0.16^*a*^
standard deviation	13.1	11.9	11.8	
median	40.5	44.0	41.0	
range	21-76	19 - 77	24 - 70	
male	21 (80.7%)	72 (66.7%)	11 (40.7%)	< 0.01^*b*,*c*^
female	5 (19.2%)	36 (33.3%)	16 (59.3%)	
Alcohol abuse	5 (19.2%)	11 (10.2%)	1 (3.7%)	0.18^b^
Cirrhosis	5 (19.2%)	19 (17.6%)	-	0.54^b^
HCC	1 (3.8%)	13 (12.0%)	-	0.47^b^
Liver fibrosis	n = 21	n = 97	n = 7	
1 or 2	19 (90.5%)	77 (79.4%)	7 (100.0%)	0.35^b^
3 or 4	2 (9.5%)	20 (20.6%)	-	

a: ANOVA; b: Chi-square; c: the percentage of female patients was significantly higher in the subgroup of inactive carriers.

*Genotypes and BCP and PC mutations* - HBV genotypes, A, D and F, were detected. Among the 161 included patients, genotype D was the most frequent (94 patients; 54%), genotype A was detected in 58 individuals (36.0%), and genotype F was detected in only nine patients (5.6%). HBV genotypes and sub-genotypes were identified by phylogenetic analysis of the sequences characterised together with reference sequences available in GenBank (file HBV_Pol_Sequences_Brazil_RP.sqn: KY809880–KY810045) (Chachá et al. 2017).

It was possible to amplify and sequence the BCP region from samples of 118 patients and the PC region from samples of 129 patients. The BCP and PC mutations more frequently detected in these subjects are shown in Tables II and IV. Among patients with HBV genotype D, the PC mutation G1896A was detected in 84.1% of the cases (53/63), while the G1862T mutation was detected in 92.3% of the subjects infected with HBV genotype A (36/39) (p < 0.01). The frequency of the A1762T/G1764A BCP double mutation was similar among the different HBV genotypes (44.2% in HBV genotype A, 40.6% in HBV genotype D, and 50% in HBV genotype F). The sequences are available in GenBank (file HBV_RP_PCC_GenBank.sqn: KY810046–KY810174).

**TABLE II:** Basal core promoter (BCP) and precore (PC) mutations frequently found in the studied sample, according to the different evolutionary stages of chronic hepatitis B virus (HBV) infection

BCP mutations n = 118	Evolutionary phase of HBV infection						p

	HBeAg+ chronic hepatitis n = 19		HBeAg-chronic hepatitis n = 76		Inactive carrier n = 23		
	n	%	n	%	n	%	
A1762T/G1764A	4	15.4	17	15.7	1	3.7	0.03^*a*^
T1753C (G or A)/ A1762T/G1764A	3	11.5	22	20.5	4	14.8	
Other BCP variants	2	7.7	17	15.7	-	-	
No BCP mutations	10	38.5	20	18.5	18	66.7	< 0.01^*b*^

Total	19	100	76	100	23	100	

PC mutations n = 129	Evolutionary phase of HBV infection						p

	HBeAg+chronic hepatitis n = 20		HBeAg-chronic hepatitis n = 85		Inactive carrier n = 24		
	
	n	%	n	%	n	%	
G1862T	7	35.0	16	18.8	5	20.8	0.28
G1896A	2	10.0	20	23.5	11	45.8	0.03^*c*^
G1896A/G1899A	1	5.0	19	22.4	-	-	
Other PC mutations	-	-	18	21.2	-	-	
No PC mutations	10	50.0	12	14.1	8	33.4	< 0.01^*d*^

Total	20	100	85	100	24	100	

a: A1762T/G1764A HBV mutation was less frequent among inactive carriers (Fisher’s exact Test); b: HBV wild-type for the BCP region (without mutations) was more frequent among inactive carriers (Qui-Square Test); c: lower frequency of G1896A HBV mutation among chronic HBeAg positive hepatitis subjects (Chi-Square Test); d: higher proportion of wild-type HBV among chronic HBeAg positive hepatitis patients (Chi-Square Test).

**TABLE IV:** Basal core promoter (BCP) and precore (PC) mutations frequently found in the studied sample, according to the presence of cirrhosis or hepatocellular carcinoma (HCC)

	With cirrhosis n = 24	Without cirrhosis n = 137	p	With HCC n = 14	Without HCC n = 147	p	Total N = 161
BCP mutations^*a*^ n =118							
A1762T/G1764A	10 (76.9%)	40 (38.1%)	0.01	7 (77.8%)	43 (39.4%)	0.03	50 (42.4%)
PC mutations^*b*^ n = 129							
G1862T	8 (50.0%)	31 (27.4%)	0.08	5 (45.4%)	34 (28.8%)	0.30	39 (30.2%)
G1896A	6 (37.5%)	50 (44.2%)	0.78	5 (45.4%)	51 (43.2%)	1.00	56 (43.4%)
G1862T/G1896A	-	1 (0.9%)	-	-	1 (0.8%)	-	1 (0.8%)

a: 118 patients were analysed (13 with cirrhosis and 105 without cirrhosis); nine with HCC and 109 without HCC; b: 129 patients were analysed (16 with cirrhosis and 113 without cirrhosis; 11 with HCC and 118 without HCC.

*BCP and PC mutations and forms of chronic HBV infection* - Among the 129 patients in whom the HBV PC region was analysed, a high frequency of HBV strains with G1896A mutation (53/129, 41%) was detected, alone or in G1896A/G1899A double mutation, particularly in the subgroup with HBeAg-negative chronic hepatitis (39/85, 45.9%) (p = 0.03). The wild type was more frequently found in the HBeAg positive group (10/20, 50%) (p < 0.01) (Table II).

The A1762T/G1764A double mutation was less frequent among inactive carriers (5/23, 21.7%) (p = 0.03) and the wild-type for the BCP region (without mutations) was more frequent among inactive carriers (18/23, 66.7%) (p < 0.01) (Table II).

*BCP and PC mutations and the stage of liver fibrosis and the presence of cirrhosis or HCC* - Liver biopsies were carried out on 125 patients. Univariate analyses were performed to assess the association between stages of fibrosis and gender, age, alcohol abuse, hepatitis B viral load, and HBV genotype. It was observed that the mean age was higher among patients with moderate or severe hepatic fibrosis (grades 3 or 4) (55.1 versus 41.2 years; p < 0.01). Regarding the other parameters, no association was observed. With regard to the presence of BCP or PC mutations, the A1762T/G1764A variant was identified in 36.7% of patients with grade 1 and 2 liver fibrosis (29/79) and in 81.8% of patients with grade 3 and 4 liver fibrosis (9/11) (p < 0.01); in 76.9% of patients with cirrhosis (10/13) and in 38.1% of patients without cirrhosis (40/105) (p = 0.01). No association was found between PC mutation and the grade of liver fibrosis.

The A1762T/G1764A variant was identified in 36.7% of patients with grade 1 and 2 liver fibrosis (29/79) and in 81.8% with grade 3 and 4 (9/11) (p < 0.01); and in 76.9% of patients with cirrhosis (10/13) and in 38.1% of patients without cirrhosis (40/105) (p = 0.01).

Among patients with cirrhosis, the percentage of males was higher. The mean age was higher among patients with cirrhosis and among those with HCC (Table III). The rate of infection with the HBV BCP A1762T/G1764A mutant was also higher in patients with cirrhosis than in patients without cirrhosis (76.9% versus 38.1%; p = 0.01). It was also observed that patients with HCC were more frequently infected with the BCP A1762T/G1764A mutant than those without HCC (77.8% versus 39.4%; p = 0.03) (Table IV).

**TABLE III:** Demographic, clinical and histological characteristics of the 161 chronic hepatitis B virus (HBV) patients evaluated according to the presence or absence of cirrhosis and hepatocellular carcinoma (HCC)

	With cirrhosis n = 24	Without cirrhosis n = 137	p	With HCC n = 14	Without HCC n = 147	p	Total N = 161
Gender			0.04			0.38	
male	20 (83.3%)	84 (61.3%)		11 (78.6%)	93 (63.3%)		104 (64.6%)
female	4 (16.7%)	53 (38.7%)		3 (21.4%)	54 (36.7%)		57 (35.4%)
Age (mean in years)	55.5	42.1	< 0.01	54.5	43.1	< 0.01	44.1
Alcohol abuse	4 (16.7%)	13 (9.5%)	0.28	3 (21.4%)	14 (9.5%)	0.17	17 (10.6%)

Multivariable logistic regression was performed to investigate independent risk factors for advanced fibrosis (grades 3 or 4), hepatic cirrhosis and HCC. Infection with the HBV BCP A1762T/G1764A mutant was associated with advanced fibrosis [odds ratio (OR) = 7.76, 95% confidence interval (95%CI) 1.57-38.39]. HCC and cirrhosis were found to be associated with the presence of the A1762T/G1764A mutant (OR = 5.42, 95% CI 1.41-20.87; and OR = 5.37, 95% CI 1.07-27.08, respectively).

## DISCUSSION AND RESULTS

HBV strains with BCP mutations, the most frequent being the A1762T/G1764A double mutation, were detected in 59.3% (70/118) of the patients studied. No association was detected between the presence of the A1762T/G1764A mutation and the different HBV genotypes. A high prevalence of HBV harbouring mutations in the PC region was observed, being detected in 80.6% of the patients. HBV with G1862T and G1896A mutations, alone or in double mutation patterns, were the most frequent (31% and 44.2%, respectively). The G1862T mutation was previously described in association with HBV genotype A, particularly with sub-genotype A1 (Kramvis et al. 1998). There was an association between the presence of the G1896A mutation and genotype D in the subjects studied here (p < 0.01). The G1896A HBV mutation is related to HBeAg negative forms of infection (Laras et al. 1998). It acts as an early stop codon, preventing *e* antigen synthesis, without interfering in the replicative capacity of the virus. The base present at position 1896 matches the 1858 base of the HBV genome. Switching base G to A at position 1896 of the genome, base T is included at position 1858. The presence of T in position 1858 is common among HBV genotype D and uncommon among HBV genotype A, so finding this type of mutation in HBV genotype A sequences is not expected unless the virus has the C1858T mutation (Li et al. 1993). The reason why there are high proportions of G1896A HBV mutations in the present study is possibly related to the high prevalence of genotype D. Among the 58 sequences classified as genotype A in this study, only one exhibited the G1896A mutation. In this viral sequence, as expected, the presence of the C1858T mutation was noted.

Some studies have associated the presence of the A1762T/G1764A mutation with a lower expression of viral antigens, *in vitro* or *in vivo* (Jammeh et al. 2008, Liu et al. 2011). In this study, no quantitative methods to detect HBsAg or HBeAg were used, so it was not possible to evaluate this parameter. However, the results showed no association between the double mutation BCP and the evolutionary phases of chronic HBV infection.

An association between infection with the HBV A1762T/G1764A BCP mutant and the presence of moderate and severe fibrosis, clinically detected hepatic cirrhosis, and the presence of HCC was verified in this study. Although there is still no consensus regarding the impact of the emergence of mutations in the HBV genome BCP region on liver disease progression, several studies have suggested that an increased virulence of HBV is related to the selection of these mutant viruses, most of them carried were out in Asia and in the HBV genotypes B and C (Kao et al. 2003, Yang et al. 2008, 2016, Zheng et al. 2011). Moreover, it was proposed that the presence of these mutations might exacerbate the host immune response, to increase viral replication and to modify the region coding for the X protein, which would ultimately be related to progression to HCC (Yang et al. 2016). In a large population-based prospective study conducted in 10 urban centres in Taiwan, in which there was a greater prevalence of infection with HBV genotypes B and C, Yang et al. (2008) detected the highest rates of progression to HCC among individuals infected by HBV carrying the A1762T/G1764A double mutation. Regarding comparisons between genotypes A and D in an Indian study, the progression of chronic hepatitis B to HCC seemed to be more frequent in patients infected by HBV genotype D and was associated with the A1762T/G1764A mutant (Asim et al. 2010). More recently, in Venezuela, Puche et al. (2016) found the BCP double mutation in early stages of HBV infection among individuals infected with genotype F2, associated with more severe forms of liver disease when compared to HBV genotype F3. The present study detected a novel association between the double BCP mutation and more severe forms of liver disease in Brazilian individuals infected with genotype A and D.

It is interesting to emphasise that other HBV variants were found after considering the BCP and PC regions. In this study, 59.3% (70 of 118) of the HBV BCP mutants and 76.7% (99 of 129) of the HBV PC mutants were noted; thus, the presence of the HBV mutant was more frequent than the wild-type virus. However, data regarding previous antiviral treatment that would be a way to induce selective pressure to escape mutants were not analysed, and this is therefore one of the limitations found in the discussion regarding the frequency of BCP and PC mutations in this case series.

Notably, although the data of the present study can be compared with those of previous studies, there are no definitions in the literature regarding the influence of genetic HBV variability on the course of chronic infection. Additionally, the design of the present study was cross-sectional and the analyses performed may not reflect the natural course of the disease in a reliable manner. Further studies, ideally conducted in a prospective manner, are necessary to confirm the present findings.

In conclusion, in the present study, a high prevalence of mutations in the PC and BCP regions of HBV was found. The presence of the HBV A1762T/G1764A BCP double mutant was independently associated with the presence of advanced forms of chronic liver disease, such as severe fibrosis, liver cirrhosis, and HCC, in Brazilian infected patients.
